# Dr. Cameron's Case of Secondary Small-Pox

**Published:** 1810-02

**Authors:** Ch. Cameron

**Affiliations:** Worcester


*To the Editors of the Medical and Physical Journal.*

Gentlemen,

THE following letter has been inserted in our papers; I am disposed to think, however, that the facts which it contains, deserve a wider circulation than can be given them by a provincial newspaper; should you be of the same opinion, you will perhaps do me the favour to insert them in your Journal.

I am, &c.

CH. CAMERON.

*Worcester, Dec.* 27, 1809.

---

AS much unfounded prejudice still exists against the cow-pox, and as inoculation for the small-pox is supposed, with undeviating certainty, to secure the constitution from any future attack of the disease: I beg leave to state the following case, which has recently occurred, as it may tend to remove the objections against the former disorder, and throw some suspicion upon the boasted security of the latter.

John Skyrme, the son of a respectable tradesman in

 this this city, 18 years of age, was on Friday, the 21st of October, seized with shivering and other symptoms of fever; on the third day, Monday, an eruption appeared, chiefly about the face and neck. When I saw it, I immediately said, this looks like the small-pox, to which his mother replied, that could not be, as he had been inoculated when he was three weeks old, together with four other children, and two servants, all of whom had the disease, to the entire satisfaction of the medical attendant, who, I may venture to assert, from my personal knowledge of him, could not have been deceived, and all the others (the children at least, of the servants, I know nothing) have hitherto escaped, which probably would not have been the event, had they not had the genuine disease. The disorder has now gone through its regular stages of suppuration and desquamation; and that no doubt might remain, I have requested several gentlemen of the profession to visit him, who are all unanimously of opinion, that he has had the small-pox. This may certainly shake our confidence in the immunity afforded by small-pox inoculation, and the following circumstance may perhaps induce us to place a superior reliance upon the cow-pox. A younger brother was ten years ago inoculated with cow-pock matter, and had the disease in a satisfactory manner, and though he slept with his sick brother every night till Monday, and has had daily communication with him since, yet he has resisted the infection, and continues in perfect health. As this is a curious and instructive statement of facts, I hope you will do me the favour to insert it in your Journal; and in order to obviate the cavils to which an anonymous statement might be liable, I shall authenticate it with the name of,

Yours, &c.

CH. CAMERON, M. B.

P. S. The younger boy still remains uninfected, at which I am not at all surprised, as subsequent to the cow-pox, he was twice inoculated with small-pox matter, which produced no effect, and he has enjoyed uninterrupted and even better health since, than he did previous to inoculation for the cow-pox.*

---

* The Editors received another copy of this communication from Mr. Dathem, of Powick.

